# Parotid gland oncocytoma: a case report

**DOI:** 10.1186/1757-1626-2-6423

**Published:** 2009-03-10

**Authors:** Efthimia Vlachaki, Apostolos Tsapas, Konstantinos Dimitrakopoulos, George Kontzoglou, Ioannis Klonizakis

**Affiliations:** 1 Second Department of Internal Medicine, Hippokratio General Hospital Aristotle University, 49 Konstantinoupoleos St, Thessaloniki, 54642, Greece; 2 Department of Ear, Nose, and Throat, Hippokratio General Hospital, 49 Konstantinoupoleos St, Thessaloniki, 54642, Greece

## Abstract

Oncocytomas are a rare group of neoplasms of the parotid gland which have been correlated to various viral infections. We report the first case of a patient with parotid oncocytoma and a previous history of chronic HBV infection.

## Introduction

Oncocytic neoplasms comprise a group of rare tumours of the parotid glands, and their incidence represents approximately 1% of parotid neoplasms [1]. Histologically they are classified according to the new World Health Organisation (WHO) classification in three distinct types, namely oncocytosis, oncocytoma and oncocytic carcinoma [2]. We herein describe the case of a 74-year old female patient with left parotid oncocytoma and a previous history of immune thrombocytopenia and chronic HBV infection.

## Case presentation

A 74-year old Greek Caucasian female patient presented to our department because of swelling of her left parotid gland during the previous one month. Her past history revealed refractory immune thrombocytopenic purpura (ITP), diagnosed six years earlier, which was treated with low dose corticosteroid (methyl-prednisolone 8 mg/day), and chronic untreated hepatitis B (HBV) infection with low viral load (HBV-DNA = 1.4 × 10^3^ IU/ml). On physical examination, the mass was elastic and mobile and the overlying skin was unaffected. Left cervical lymphadenopathy was detected. There were no facial palsy, xerophthalmia and/or xerostomia (Sicca syndrome).

**Figure 1:**
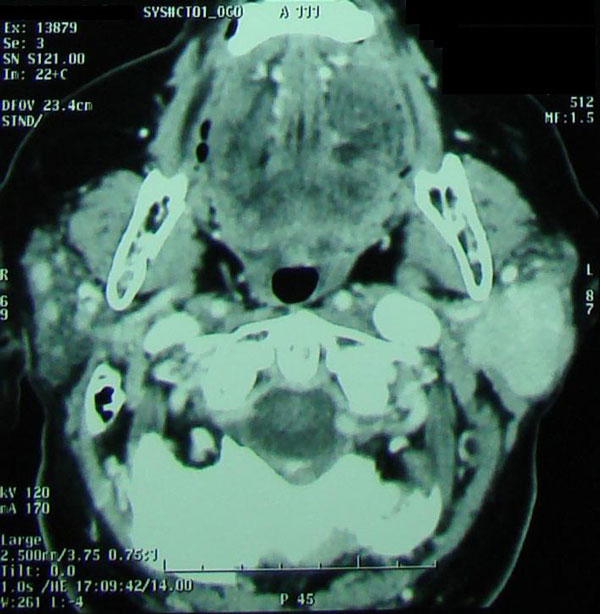
**Computer tomography (CT) findings: tumour mass of the left parotid gland**.

Extensive screening for viruses (HCV, HIV, EBV, CMV, ECHO, Coxsackie and Adenoviruses) and autoimmune diseases proved negative. Computed tomography (CT) of the parotid glands, neck and thorax revealed an egg-shaped mass in the left parotid gland (maximum diameter 3.8 cm) along with cervical lymphadenopathy (Figure 1). A biopsy with fine needle aspiration was not diagnostic and the patient underwent radical left parotidectomy. Histologic examination showed epithelial cell proliferation; cells were characterised by small round nuclei and microgranular, eosinophilic cytoplasm. A mitotic count was negative. The mass was surrounded by a thin fibrous capsule. These findings were consistent with oncocytoma of the parotid gland.

No complications were recorded during the post-operative course. During the follow-up period (12 months) no cervical lymph nodes were detected on repeated CTs and the patient remains disease free.

## Discussion

Oncocytomas usually occurr in the elderly and affect the parotid glands in 80% [3]. Bilateral oncocytoma is reported to be extremely rare, accounting for 7% of these cases [4]. Diagnosis is assisted by CT and/or magnetic resonance imaging (MRI) of the neck, although histopathologic confirmation is necessary. However, in a recent report, CT findings were correlated to histopathologic features [5].

Pathologically, oncocytoma is described as a well circumscribed mass, composed of layers of oncocytes (small round nucleus, micro-granular, eosinophilic cytoplasm). Fine needle aspiration is the procedure of choice for making a diagnosis in the majority of cases, although its sensitivity is reported to be only 29% [3]. Rarity of the disease, sampling error and lack of interpreter experience account for the majority of pitfalls.

Pathogenesis is quite obscure, although mitochondrial functional defects are believed to mediate the progressive degeneration of the salivary epithelial cells [3]. Of note, only one mitochondrial DNA rearrangement (among 200 described) has been linked to parotid tumorogenesis [6]. The correlation of certain viruses, such as EBV, HIV, HHV-8, HTLV-1 and HPV with parotid neoplasias has been documented [7]-[9]. However, there is no evidence for a possible link between HBV and these neoplasias. The immune dysregulation associated with chronic HBV infection and long-term steroid therapy may be responsible for the developed oncocytoma in our patient.

Surgical management with radical or superficial parotidectomy represents the cornerstone of therapy [3]. Probably, there is no need for chemotherapy and/or irradiation, given the benign nature and slow growth rate of the tumour; recurrence is less than 20%, mainly because of incomplete surgical resection.

## Conclusion

Oncocytic neoplasms should be considered as a possible diagnosis in patients with parotid enlargement. Due to the lack of large series, assiduous study of the cases reported in the literature may lead to better understanding of this rare disease.

## Abbreviations

CMV: Cytomegalovirus; EBV: Epstein-Barr virus; ECHO: Enteric Cytopathic Human Orphan virus; HBV: Hepatitis B virus; HCV: Hepatitis C virus; HHV-8: Human Herpesvirus 8; HIV: Human immunodeficiency virus; HPV: Human papillomavirus; HTLV-1: Human T-lymphotropic virus type 1; ITP: Immune thrombocytopenic purpura; MRI: Magnetic resonance imaging; WHO: World Health Organization.

## Consent

Written informed consent was obtained from the patient for publication of this case report and accompanying image. A copy of the written consent is available for review by the Editor-in-Chief of this journal.

## Competing interests

The authors declare that they have no competing interests.

## Authors' contributions

EV analyzed and interpreted the patient data regarding the haematological disease and was a major contributor in the literature review and the critical revision of the manuscript. AT reviewed the relative literature and was the major contributor in writing and critically revising the manuscript. KD and IK analyzed and interpreted the patient data and reviewed the relative literature. GK was the main surgeon and reviewed the relative literature. All authors have read and approved the final version of the paper.
